# Validity and reliability of the French version of the quick‐FAAM (Q‐FAAM‐F) among patients undergoing anatomic ankle ligament reconstruction

**DOI:** 10.1002/jeo2.70644

**Published:** 2026-01-19

**Authors:** Ibrahim Saliba, Olivier Grimaud, Vincent Fontanier, Brice Picot, Frederic Khiami, Gregoire Rougereau, Yoann Bohu, Nicolas Lefevre, Alexandre Hardy

**Affiliations:** ^1^ Orthopedic Surgery Department Cochin Hospital Paris France; ^2^ Orthopedic Surgery Department Clinique Du Sport Paris France; ^3^ Medinetic Learning Paris France; ^4^ Inter‐University Laboratory of Human Movement Biology (LIBM) Savoie Mont‐Blanc University Chambéry France; ^5^ French Society of Sports Physical Therapy (SFMKS Lab) Pierrefitte‐sur‐Seine France; ^6^ Orthopedic Surgery Department, Pitié‐Salpêtrière Hospital Sorbonne University Paris France

**Keywords:** anatomic ankle ligament reconstruction, ankle instability, French version of quick‐FAAM, French‐speaking patients, quick‐FAAM

## Abstract

**Purpose:**

To evaluate the validity and reliability of the French version of the Quick Foot and Ankle Ability Measure (Q‑FAAM‑F) in French‑speaking patients with chronic lateral ankle instability (CLAI).

**Methods:**

We conducted a prospective observational cohort in a sports surgery centre with repeated assessments preoperatively, and at 3 and 6 months postoperatively; the primary analysis was cross‑sectional at 6 months. Consecutive CLAI patients undergoing anatomic lateral ankle ligament reconstruction (AALR) were included. Patients completed the Q‑FAAM‑F (12 items derived from the validated French FAAM) alongside the full FAAM, FAOS, ALR‑RSI, CAIT and VAS‑pain. Internal consistency (Cronbach's *α*), item–total and inter‑item correlations, and construct validity (Pearson's *r*) were calculated. Discriminant validity used ROC analyses for CLAI status (CAIT < 24) and return to sport (RTS), defined on the RTS continuum as return to the pre‑injury sport at any level at 6 months and treated as an external clinical variable.

**Results:**

Among 275 patients (56% male; median age 32 years), Q‑FAAM‑F showed excellent internal consistency (*α* = 0.96) and strong item–total correlations (mean *r* ≈ 0.65). Convergent validity was strong with the FAAM (*r* = 0.95) and with FAOS and ALR‑RSI; divergent validity was supported by the absence of correlation with CAIT of the nonoperated limb. ROC AUC for CLAI status and RTS were high; optimal cut‑offs were 78.1/100 (CLAI: sensitivity 81.3%, specificity 85.4%) and 80.2/100 (RTS: sensitivity 75.4%, specificity 87.9%).

**Conclusion:**

The Q‑FAAM‑F is a valid and reliable PROM for French‑speaking CLAI patients, suitable for clinical practice and research. Precise AUC‑based thresholds may support clinical decision‑making at 6 months.

**Level of Evidence:**

Level III.

**Abbreviations**
AALR: anatomic ankle ligament reconstruction
ALR‐RSI: ankle ligament reconstruction–return to sport after injury
ATFL: anterior talo‐fibular ligament
AUC: area under the curve
CAIT: Cumberland ankle instability tool
CFL: Calcaneo‐fibular ligament
CLAI: chronic lateral ankle instability
FAAM: foot and ankle ability measure
FAOS: foot and ankle outcome score
PROM: patient‐reported outcome measure
Q‐FAAM‐F: French quick‐FAAM
*r*
: Pearson correlation coefficient
ROC: receiver operating characteristic
RTS: return to sport
VAS: visual analog scale

## INTRODUCTION

Evaluation of patients with musculoskeletal disorders can utilise not only clinical examination, functional tests or radiological imaging but also patient‐reported outcome measures PROMs [4, 9]. The value of information obtained from these instruments depends on evidence supporting the interpretation of their scores [5, 24]. The foot and ankle ability measure (FAAM), originally developed in English, has demonstrated reliability, validity and responsiveness as a measure of physical function [25]. These psychometric properties have been established in individuals with a wide range of musculoskeletal disorders affecting the lower leg, ankle and foot, making it broadly applicable [5, 25]. To assess activities requiring higher functional ability, the FAAM also includes a Sports subscale [23].

Specific interpretive parameters, such as the minimal detectable change (MDC) and the minimal clinically important difference (MCID), have been provided for the FAAM [4, 9]. Its utility is supported for various populations, including athletes with chronic ankle instability [6] and patients with diabetes mellitus [23]. Additionally, cross‐cultural adaptations and validations have resulted in the development of German [28], Persian [27] and French [5] versions of the FAAM. Among more than 50 existing patient‐reported outcome measures for individuals with chronic lateral ankle instability (CLAI), FAAM has demonstrated the most rigorous validation and superior measurement properties, according to Hansen et al. [13], and might therefore be preferred by clinicians for the assessment of CLAI.

While the FAAM has robust psychometric properties [18]—including test‐retest reliability, internal consistency, validity and responsiveness—and is widely used for diverse foot and ankle conditions, concerns exist regarding its efficiency [19, 20, 26]. The time required to administer both FAAM subscales is notable (i.e., 21 items for the activities of daily living (ADL) and 8 for the sports subscale, respectively), with prior studies reporting that the ADL subscale requires slightly less time than the Foot Function Index, despite having fewer items [19, 20]. Furthermore, both subscales are significantly more time‐intensive than computerised adaptive testing methods [18]. Authors in prior studies determined that several items on the FAAM were not relevant to patients with CLAI [5, 27, 28]. To improve efficiency, the Quick‑FAAM was developed as a 12‑item single scale preserving psychometrics while reducing burden.

To address these limitations, a reduced English version of the FAAM, referred to as the Quick‐FAAM, has been developed [16, 17, 18]. This version was designed to reduce the number of items while preserving the instrument's strong psychometric properties, enhancing its utility in clinical and research settings [16, 17, 18]. The Quick‐FAAM condenses the original 29 items into a 12‐item single scale, retaining 7 items from the Sports subscale and 5 items from the ADL subscale [18]. This modification aims to improve efficiency and relevance, particularly for patients with CLAI.

Although there is increasing evidence to support the use of the Quick‐FAAM [15], it has not been adapted and validated for French‐speaking individuals. In our clinical and research practice, most patients are French‐speaking and not able to appropriately respond to items written in English. The aim of this study was to provide evidence for validity and reliability for the French version of the Quick‐FAAM (Q‐FAAM‐F).

## METHODS

### Development of the Q‐FAAM‐F

**Figure 1:**
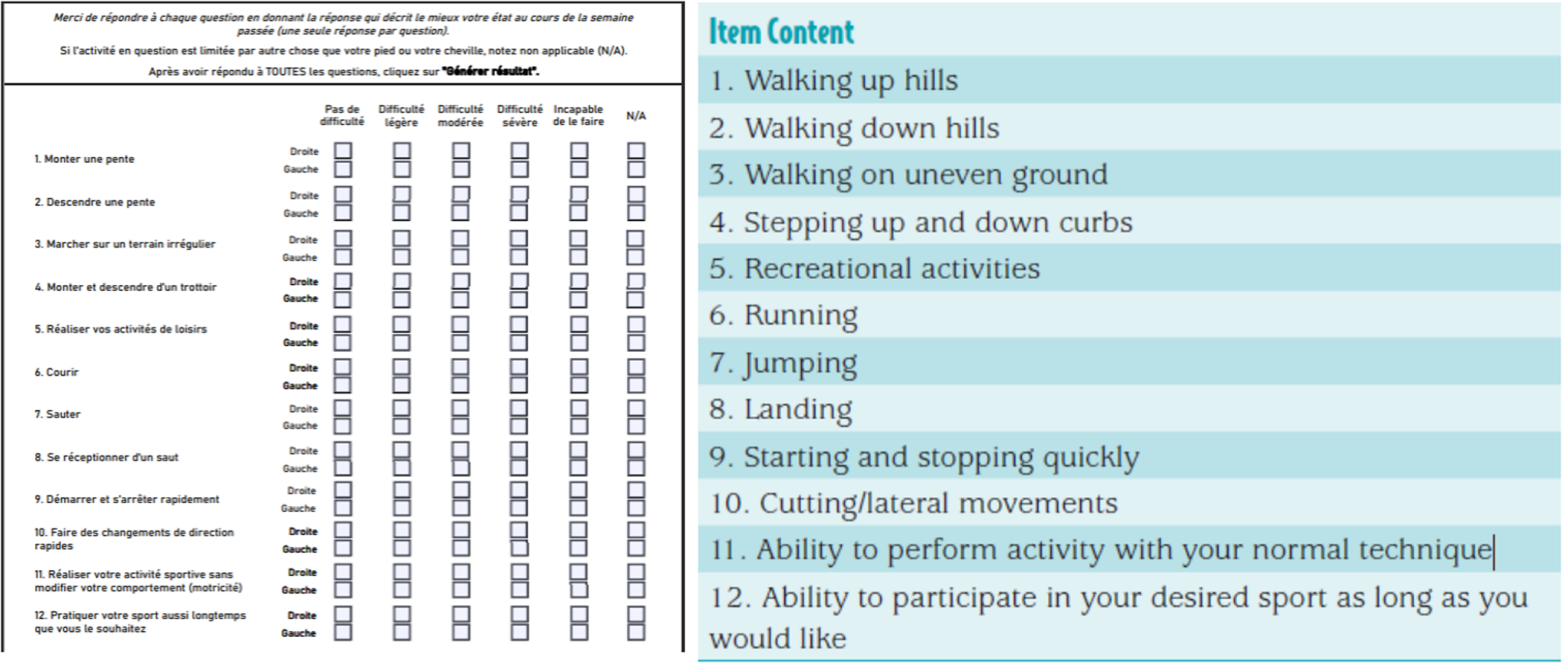
The French Q‑FAAM‑F (12 items) with parallel English item stems [18] displayed alongside the validated French wording to facilitate international readership.

The cross‐cultural adaptation of the original FAAM into French [5] was produced via forward‐back translation in accordance with the guidelines of the American Academy of Orthopedic Surgeons (AAOS) Outcomes Committee, as recommended in the literature [1, 3]. The French adaptation of the FAAM was published in 2011 [5]. The Quick‐FAAM, first introduced by Hoch et al. in 2016 [18], has been validated as a reliable instrument. In our study, the Q‑FAAM‑F was created by extracting the 12 Quick‑FAAM items from the validated French FAAM wording (Figure 1), reviewed by a bilingual panel (two orthopaedic surgeons, one sports physio and one PROMs methodologist) to ensure conceptual equivalence with the English Quick‑FAAM and linguistic clarity for metropolitan and nonmetropolitan French. No de novo translation was required.

### Study design and population

#### Study design and setting

Prospective observational cohort with repeated measures at pre‑op, 3 months and 6 months post‑op; the primary analytic approach was cross‑sectional using the 6‑month timepoint. The study was conducted at a dedicated sports surgery centre.

#### Ethics

Approved by the local institutional review board with authorisation No.: COS‐RGDS‐2025‐07‐005‐HARDY‐A; IRB number (IRB00010835); all patients provided informed consent.

#### Participants

**Figure 2:**
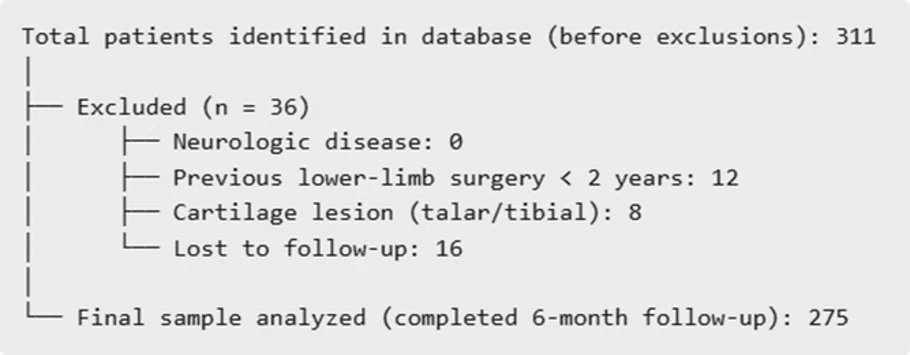
STROBE flowchart patient selection process.

Consecutive patients with CLAI undergoing anatomic ankle ligament reconstruction (AALR) between January 2019 and June 2024 were identified from the institutional database using diagnosis/procedure codes. Exclusion criteria: neurological disease; lower‑limb surgery within 2 years prior to AALR (to avoid confounding functional recovery from a recent surgery); and osteochondral/cartilage lesions of talus or tibia. A STROBE‑conformant flowchart summarising selection, exclusions and analysed samples is provided (Figure 2).

### Outcome measure and variables

Patients completed electronic versions of the French‐FAAM, Q‐FAAM‐F and French versions of the foot and ankle outcome score (FAOS) [32], ankle ligament reconstruction‐return to sport index (ALR‐RSI) [29, 34], Cumberland Ankle Instability Tool (CAIT) [10] and a visual analog scale (VAS) for pain (VAS context: pain during usual daily activities at rest and with activity averaged over the past week.) [8, 22]. In addition to functional scores, patients were asked at each follow‐up to complete the return to sport (RTS) data. Consistent with the RTS continuum [2], RTS at 6 months was recorded via a binary item (‘returned to the same sport at any level: Yes/No’) and treated as an external clinical anchor for discriminative analyses, not part of the FAAM scoring. The binary RTS item was used as an external criterion on the RTS continuum (return to participation → sport → performance) to evaluate discriminative validity; it was not included in Q‑FAAM‑F scoring.

### Surgical technique

All patients underwent a standardised surgical procedure involving direct reconstruction of the anterior talofibular ligament (ATFL) and calcaneofibular ligament (CFL) using gracilis tendon autografts [21]. The grafts were harvested from the ipsilateral knee. Contralateral harvest was avoided to prevent donor morbidity to the healthy limb; allograft was used only when prior ipsilateral harvest precluded autograft use. Under arthroscopic guidance, the graft was fixed distally in a talar tunnel for ATFL reconstruction and a calcaneal tunnel for CFL reconstruction. Proximal fixation to the fibula was achieved by passing the graft through a fibular tunnel.

### Postoperative protocol

Postoperative care included immediate weight‐bearing in a walking boot for 3 weeks. Patients followed a tailored rehabilitation protocol based on individuals impairments [7]. was permitted after Ankle‑GO™ score > 15 per published guidance [14].

### Statistical analyses

#### 1‐Reliability and concurrent validity

Reliability and concurrent validity of the Q‐FAAM‐F were assessed using the following methods:

##### Descriptive statistics and distribution analysis

Item‐level and total score distributions were examined for normality and skewness.

Descriptive statistics were calculated for each item and the total score. We report mean ± SD for approximately normal variables and median (interquartile range [IQR]) for nonnormal variables, with categorical data as *n* (%).

Skewness values ≥ ± 1.96 would indicate that subjects consistently scored at the floor or ceiling for an item, while a corrected item‐total correlation of ≤ 0.30 would indicate an item is answered inconsistently in respect to all other items on the instrument, which is represented by the total score [18].

##### Internal consistency

Internal consistency of the questionnaire was evaluated using Cronbach's alpha.

##### Inter‐item correlations

Correlations between individual items were calculated to assess the relationships between items.

##### Item‐total correlations

Correlations between each item and the total FAAM score (excluding the contribution of the item in question) were computed.

#### 2‐Convergent and divergent validity

Convergent and divergent validity was examined by calculating the correlations between the Q‐FAAM‐F score and scores from validated instruments, including: ALR‐RSI, FAOS (Total score as well as its subscales for pain, stiffness and quality of life), Full FAAM score (Total score as well as its subscales for activities of daily life and sports), VAS and CAIT.

Convergent validity was examined through Pearson correlations with the abovementioned scores. The Pearson correlation coefficients (*r*) used for establishing validity were interpreted as weak (0–0.40), moderate (0.41–0.69), or strong (0.70–1.00). The coefficient of determination (*r* ^2^) was also calculated to examine the explained variance exhibited for each analysis. Alpha was set at *p* ≤ 0.05 for all analyses.

#### 3‐Discriminant validity

Discriminant validity was evaluated through receiver operating characteristic (ROC) curve analyses for two functional outcomes:

Presence of CLAI: Patients were categorised as CLAI (CAIT < 24) or non‐CLAI (≥ 24 points) [12].

RTS: Assessed through patient‐reported RTS data by a ‘Yes or No’ question.

The following metrics were reported from ROC analyses:

Area under the curve (AUC): A measure of the diagnostic performance of the Q‐FAAM‐F.

Sensitivity and specificity: To evaluate the questionnaire's ability to correctly classify patients.

Optimal cut‐off: Determined using Youden's Index: [35] defined as the threshold maximising the distance from the identity line [35]; it was mathematically determined as the value that maximises the sum of sensitivities and specificities.

### Software and tools

All statistical analyses were performed using the software version 3.5.0, which can be accessed at the URL https://www.R-project.org.

Specific R packages were utilised as follows:

dlookr: [33] For descriptive statistics and distribution analysis.

Psych: [30] For internal consistency estimates.

pROC: [31] For ROC curve analyses.

Sample size: No a priori sample size calculation was performed.

Reliability extensions (not performed). Test–retest reliability (ICC), standard error of the mean (SEM) and MDC were not evaluated in this study because repeated stable‑state measurements were not collected; these are identified as priorities for future work.

Structural validity (not performed). Factor/Rasch analyses were beyond scope; we discuss implications in the Discussion.

## RESULTS

### Patient demographics

The study included 275 consecutive patients, comprising 155 males and 120 females, with a median age of 32 years (range: 14–72 years). Detailed demographic and descriptive characteristics of the study population are summarised in Table 1. The participant flowchart is shown in Figure 2.

**Table 1:** This table summarises patients' demographic characteristics (patients characteristics *N* = 275).

Characteristic	Value
Sex, *n* (%)	Male 154 (56.0), female 121 (44.0)
Age (years)	Median (IQR); 32 (14–72)
Height (cm)	Median (IQR); 174 (168–200)
Weight (kg)	Median (IQR); 74 (42–107)
Sport practice, *n* (%)	Professional 28 (10.2), Competitive 91 (33.1), Regular 106 (38.5), Occasional 50 (18.2)
Sport type, *n* (%)	Pivot‐contact 111 (40.4), Pivot 91 (33.1), Linear 73 (26.5)

### Descriptive statistics and distribution analysis

**Figure 3:**
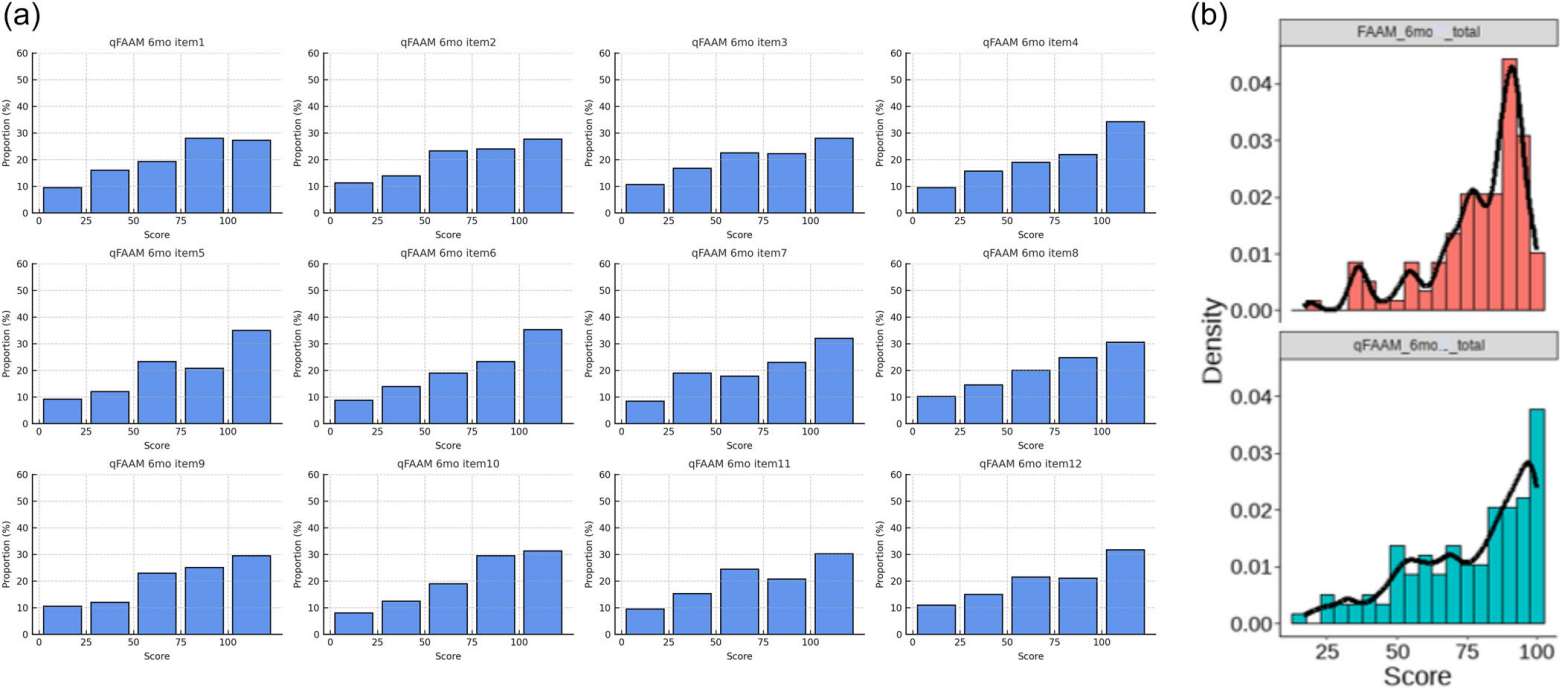
(a) The analysis of the data revealed that the distribution of scores for most items in the quick Q‐FAAM‐F was symmetric. (b) The overall distribution of Q‐FAAM‐F scores aligns closely with that of the total FAAM, with no substantial abnormalities observed in density plots for the total scores. mo, months; qFAAM, Q‐FAAM‐F.

The analysis of the data revealed that the distribution of scores for most items in the quick Q‐FAAM‐F was symmetric and without significant anomalies. However, Item 4 showed an asymmetric distribution with a ceiling effect, as reflected in its higher mean score (94.35 ± 13.27) and a negative skewness (−2.29). Despite this isolated observation, the overall distribution of Q‐FAAM‐F scores (mean ± SD: 75.86 ± 21.68) aligns closely with that of the total FAAM (78.73 ± 17.57), with no substantial abnormalities observed in the skewness or density plots for the total scores (Figure 3 and Table 2).

**Table 2:** This table shows the analysis of scores for the items of the Q‐FAAM‐F. The distribution of these scores was symmetric and without significant anomalies. However, Item 4 showed an asymmetric distribution with a ceiling effect, as reflected in its higher mean score (94.35 ± 13.27) and a negative skewness (−2.29). The overall distribution of Q‐FAAM‐F scores aligns closely with that of the total FAAM.

Item	Mean ± SD	Skewness
item1	82.11 ± 22.80	−1.21
item2	82.11 ± 23.03	−0.99
item3	78.29 ± 23.17	−0.80
item4	94.35 ± 13.27	−2.29
item5	78.54 ± 24.75	−0.84
item6	71.59 ± 28.63	−0.65
item7	71.02 ± 30.25	−0.71
item8	67.70 ± 29.63	−0.62
item9	75.22 ± 27.09	−0.85
item10	77.83 ± 26.85	−1.16
item11	67.95 ± 30.12	−0.55
item12	62.61 ± 34.96	−0.42
qFAAM_6mo_total	75.86 ± 21.68	−0.78
FAAM_6mo_total	78.73 ± 17.57	−1.28

Abbreviations: mo, months; qFAAM, the French version of the Quick FAAM questionnaire; SD, standard deviation.

Moreover, the correlation analysis between each item of the Q‐FAAM‐F and the total FAAM score (corrected and uncorrected) showed moderate to strong correlations, ranging from 0.66 to 0.82 (Table 3). These findings demonstrate a consistent convergent validity between the items of the Q‐FAAM‐F and the total FAAM score, indicating that both measures capture similar constructs with a high degree of consistency.

**Table 3:** This table shows the correlation analysis between each item of the Q‐FAAM‐F and the total FAAM score (corrected and uncorrected). There are moderate to strong correlations, ranging from 0.66 to 0.82.

Item	[*r*]	[*t*]	*p*	[df]	[*r*²]
item1	0.81	14.71	4.18e‐28	114	0.65
item2	0.73	11.28	2.84e‐20	114	0.53
item3	0.81	14.58	1.23e‐27	112	0.65
item4	0.67	9.71	1.42e‐16	113	0.45
item5	0.74	11.48	1.37e‐20	111	0.54
item6	0.82	15.01	3.39e‐28	108	0.68
item7	0.79	13.81	7.61e‐26	111	0.63
item8	0.79	13.45	4.72e‐25	111	0.62
item9	0.68	9.82	7.50e‐17	114	0.46
item10	0.66	9.38	8.20e‐16	113	0.44
item11	0.73	11.03	2.13e‐19	108	0.53
item12	0.74	11.43	2.98e‐20	107	0.55
[uncorrected_qFAAM]	0.95	32.28	1.12e‐55	103	0.91
[corrected_qFAAM]	0.81	13.85	2.96e‐25	103	0.65

Abbreviations: corrected_qFAAM, adjusted or normalised composite score from the Q‐FAAM‐F questionnaire; df, degrees of freedom; mo, months; qFAAM, the French version of the Quick‐FAAM questionnaire Q‐FAAM‐F; *r*, Pearson correlation coefficient; *r*², coefficient of determination (explained variance); *t*, *t*‐statistic from correlation analysis; uncorrected_qFAAM, raw/uncorrected composite score from the Q‐FAAM‐F questionnaire.

### Average inter‐item correlation and internal consistency

The analyses revealed inter‐item redundancy, with an average correlation of *r* = 0.65. Additionally, the internal consistency was excellent, as indicated by a Cronbach's alpha of *α* = 0.96.

### Convergent validity

The convergent validity analysis showed a strong correlation between the Q‐FAAM‐F and the FAAM (*r* = 0.95) as well as with other questionnaires, including the FAOS, ALR‐RSI, VAS and CAIT of the operated limb. In contrast, no correlation was observed with the CAIT of the nonoperated limb, indicating excellent convergent and divergent validity (Table 4).

**Table 4:** The convergent validity analysis showed a strong correlation between the Q‐FAAM‐F and the FAAM (*r* = 0.95) as well as with other questionnaires, including the FAOS, ALR‐RSI, VAS and CAIT of the operated limb. In contrast, no correlation was observed with the CAIT of the nonoperated limb.

Item	[*r*]	[*t*]	*p*	df	[*r*²]
ALR_6mo_total	0.74	10.62	9.93e‐18	93	0.55
FAOS_6mo_Pain	0.86	16.24	6.90e‐29	93	0.74
FAOS_6mo_Stiffness	0.78	11.97	1.57e‐20	93	0.61
FAOS_6mo_qol	0.88	18.13	2.74e‐32	93	0.78
FAOS_6mo_total	0.90	19.79	4.24e‐35	93	0.81
FAAM_6mo_sports	0.98	56.18	4.31e‐79	103	0.97
FAAM_6mo_activites	0.85	16.49	1.18e‐30	103	0.73
FAAM_6mo_total	0.95	32.28	1.12e‐55	103	0.91
EVA_6mo_total	−0.73	−10.04	2.38e‐16	90	0.53
CAIT_6mo_op	0.77	12.32	5.58e‐22	103	0.60
CAIT_6mo_non_op	0.08	0.82	4.11e‐01	103	0.01

Abbreviations: df, degrees of freedom; mo, months; non_op, nonoperated ankle; op, operated ankle; qol, quality of life, *r*, Pearson correlation coefficient; *r*², coefficient of determination (explained variance); *t, t*‐ statistic from correlation analysis.

### Discriminant validity regarding the presence of CAI stability

**Figure 4:**
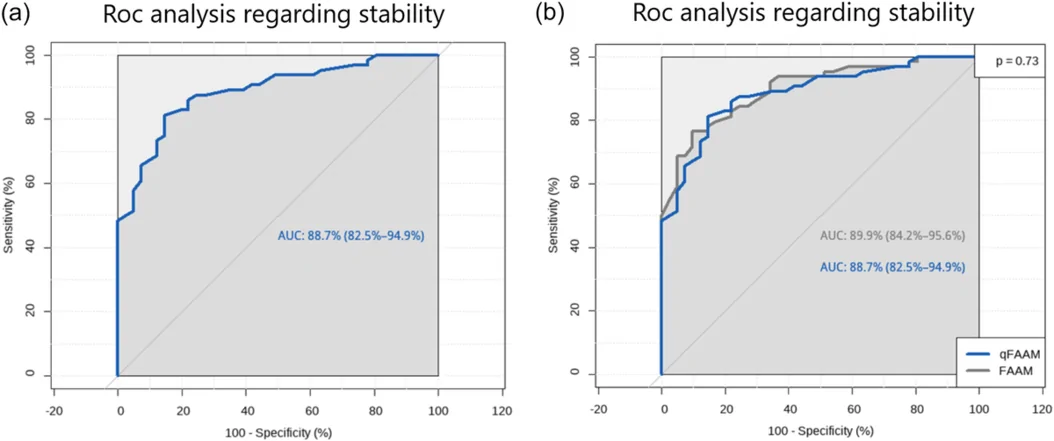
(a) The discriminative performance analysis regarding stability showed strong results, with the AUC indicating high discrimination. For the Q‐FAAM‐F at 6 months, the optimal cut‐off was 78.1 points out of 100, with a sensitivity of 81.3% and a specificity of 85.4%. (b) the discriminative validity analysis comparing the Q‐FAAM‐F and FAAM for stability at 6 months showed that there is no significant difference in the diagnostic performance between the Q‐FAAM‐F and FAAM, indicating that their ability to assess stability at 6 months is of a similar magnitude. AUC, area under the curve; qFAAM, Q‐FAAM‐F; ROC, receiver operating characteristic.

The discriminative performance analysis showed strong results, with the AUC indicating high discrimination. The statistical power of the analysis was *β* = 0. For the Q‐FAAM‐F at 6 months, the optimal cut‐off was 78.1 points out of 100, with a sensitivity of 81.3% and a specificity of 85.4% (Figure 4a). These findings support the discriminative validity of the Q‐FAAM‐F at 6 months in assessing stability, defined as a CAIT score greater than 24 [11].

Furthermore, the discriminative validity analysis comparing the Q‐FAAM‐F and FAAM for stability at 6 months showed strong discriminative performance for both measures. No significant difference was observed in the diagnostic performance between the Q‐FAAM‐F and FAAM, indicating that their ability to assess stability at 6 months is of a similar magnitude (Figure 4b).

### Discriminant validity regarding RTS

**Figure 5:**
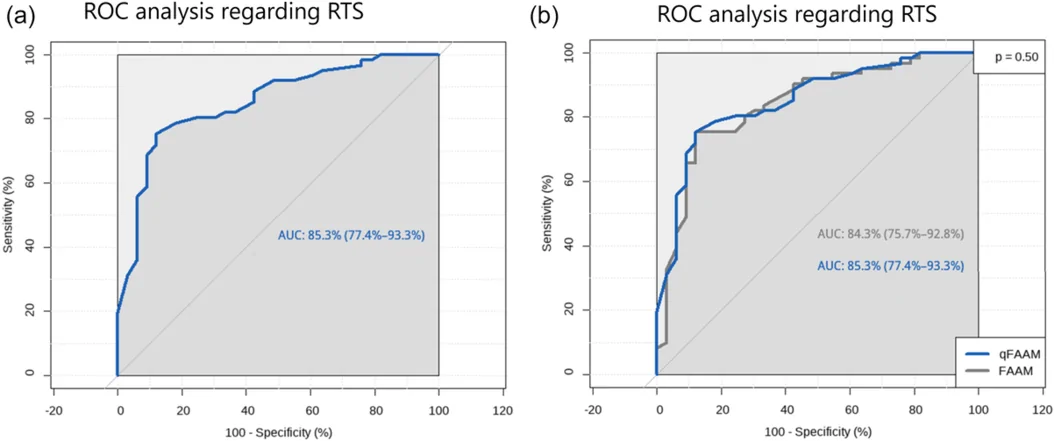
(a) The discriminative validity analysis regarding RTS showed strong discriminative performance, as indicated by the AUC (85.3% [77.4%–93.3%]). The optimal cut‐off point for the Q‐FAAM‐F was 80.2 points out of 100, with a sensitivity of 75.4% and a specificity of 87.9%. (b) The discriminative validity analysis comparing the Q‐FAAM‐F and FAAM for RTS at 6 months showed strong discriminative performance for both measures. No significant difference was observed between the Q‐FAAM‐F and FAAM, indicating that their ability to assess RTS at 6 months is of a similar magnitude. AUC, Area under the curve; qFAAM, Q‐FAAM‐F; ROC, receiver operating characteristic.

The discriminative validity analysis for RTS, regardless of the level but to the same sport at 6 months, showed strong discriminative performance, as indicated by the AUC (85.3% [77.4%–93.3%]). The statistical power of the analysis was very high (β < 0.1%). The optimal cut‐off point for the Q‐FAAM‐F was 80.2 points out of 100, with a sensitivity of 75.4% and a specificity of 87.9% (Figure 5a). These results indicate that the Q‐FAAM‐F has strong discriminative validity for assessing RTS at 6 months. Furthermore, the discriminative validity analysis comparing the Q‐FAAM‐F and FAAM for RTS at 6 months showed strong discriminative performance for both measures. No significant difference was observed between the Q‐FAAM‐F and FAAM, indicating that their ability to assess RTS at 6 months is of a similar magnitude (Figure 5b).

## DISCUSSION

The findings of this study demonstrate that the Q‐FAAM‐F shows robust psychometric properties, supporting its validity and reliability as a measurement tool. The internal consistency of the Q‐FAAM‐F was excellent, with strong inter‐item correlations and moderate to strong correlations between individual items and the total FAAM score. Convergent validity was confirmed through high correlations with the FAAM and other relevant questionnaires, while divergent validity was evidenced by the absence of correlation with the CAIT for the nonoperated limb. The discriminative validity of the Q‐FAAM‐F was also established, with strong performance in distinguishing between CLAI and non‐CLAI participants and in RTS at 6 months. Notably, the Q‐FAAM‐F diagnostic performance for stability was comparable to that of the full FAAM.

In comparing this study results with those of Hoch et al. [18], we observed no significant differences in the distribution of scores, as indicated by the absence of items with skewness values exceeding the ± 1.96 threshold, suggesting symmetrical data. Specifically, the total scores for the Q‐FAAM‐F and FAAM in our cohort were 75.86% ± 21.68% and 78.73% ± 17.57%, respectively, which are comparable to the findings reported by Hoch et al. [18], where the English Quick‐FAAM and the original English FAAM total scores were 70.31 ± 16.60% and 81.21 ± 14.07%. However, it is worth noting that Item 4 in our study showed a ceiling effect at 6 months, consistent with earlier recovery of basic ADL in postoperative CLAI cohorts; this may limit discrimination among higher‑functioning patients at later stages. Future assessments at > 6–12 months and in athletic subgroups could clarify responsiveness and potential item targeting. Despite this, the overall distribution of the data did not reveal any abnormal patterns, aligning with the expectations of typical response distributions in the context of the instruments used.

In line with the findings of Hoch et al. [18], where no items were removed due to weak corrected item‐total correlations (all *r*‐values ≥ 0.61), our study also demonstrated moderate to strong correlations between each item and the FAAM, with *r*‐values ranging from 0.66 to 0.82. These results suggest robust convergent validity between the items of the Q‐FAAM‐F and the FAAM, consistent with the correlation patterns observed in the English validation of the Quick‐FAAM.

Consistent with the findings of Hoch et al. [18], which reported acceptable redundancy across items (average *r* = 0.59) and excellent internal consistency (*α* = 0.94), the present study also demonstrated similar inter‐item redundancy, with an average *r*‐value of 0.65. Furthermore, our study revealed excellent internal consistency (*α* = 0.96), along with other indicators of reliability. These results align closely with those reported in the English version of the Quick‐FAAM [15, 16, 17, 18].

Furthermore, discriminant validity analyses led to determine a cut‐off in this study for Q‐FAAM‐F regarding the presence of CAI and level of RTS, which may be beneficial in clinical practice. Regarding discriminant validity, we prespecified that CAIT of the nonoperated limb should show negligible correlation with Q‑FAAM‑F since it taps perceived instability of an unaffected ankle; the observed noncorrelation supports discriminant properties.

From all the above, we see that the psychometric properties of Q‐FAAM‐F align with those of the English version of Quick‐FAAM, making it a valid and reliable instrument to be used in French‐speaking patients.

Regarding clinical relevance, the derived thresholds (≈ 78/100 for CAI status; 80/100 for RTS at 6 months) provide actionable markers for postoperative review. Patients scoring below these values may warrant targeted rehab, psychological readiness assessment (ALR‑RSI), or delayed RTS clearance.

Regarding limitations, although the study design is prospective with repeated measures, our primary analysis is cross‑sectional at 6 months, limiting longitudinal inferences about change. Additionally, the external validity of our findings may be questioned due to the specific patient population included in the study, as it primarily involved individuals with ankle instability who underwent ankle ligament reconstruction. This focused sample may limit the generalisability of the results to other patient populations or those with different foot and ankle pathologies. However, despite this limitation, the Q‐FAAM‐F demonstrated strong correlations with established outcome measures, such as the FAAM, FAOS, ALR‐RSI and CAIT, which are widely used in various foot and ankle disorders. These findings support the potential applicability of the Q‐FAAM‐F to broader clinical contexts, even though its validation in more diverse populations is warranted. Regarding structural validity, no structural validity (factor/Rasch) analyses were done. Given that the Quick‑FAAM is a reduced item set intended to reflect a single latent construct, future work should evaluate unidimensionality (e.g., CFA with fit indices or Rasch analysis) and local dependence. This would complement classical test statistics and inform potential item re‑targeting in French. Moreover, no a priori sample size calculation. We acknowledge this as a limitation. The achieved sample (*N* = 275) provides narrow CIs around *α* and large‑effect correlations (e.g., *r* ≥ 0.5) and adequate precision for AUC estimates.

No test–retest reliability was performed in a clinically stable window because repeated stable‑state measurements were not collected. Therefore, ICC, SEM and MDC could not be estimated. These are identified as priorities for future work. Since results of the current studies are really similar to those obtain from Hoch et al. [15, 16, 17, 18], it might be reasonable to comparable test‐retest test‐retest reliability (ICC = 0.82).

Concerning generalisability, this is a single‑centre cohort of postoperative CLAI patients; not generalisable to children or nonoperative cohorts without further validation. Although conducted at a single French centre, the language adaptation and inclusive eligibility support applicability to French‑speaking populations broadly.

Lastly, further investigations should be conducted to determine the cut‐off to discriminate between ankle sprain copers and CAI patients. Results from Hoch et al. 2020 revealed that the cutoff score differentiating between the groups was 94.79%.

## CONCLUSION

The Q‑FAAM‑F is a valid and reliable PROM for French‑speaking patients after AALR for CLAI. At 6 months, the instrument shows: Cronbach's *α* = 0.96; convergence with FAAM (*r* = 0.95); and discriminant thresholds of 78/100 for CAI status (Se 81.3%, Sp 85.4%) and 80/100 for RTS (Se 75.4%, Sp 87.9%). These precise values may assist clinical monitoring and RTS decision‑making.

## AUTHOR CONTRIBUTIONS

**Ibrahim Saliba**: Conceptualisation; data curation; methodology and writing original draft. **Olivier Grimaud**: Methodology; validation. **Vincent Fontanier**: Data curation; investigation; software. **Brice Picot**: Writing—review and editing; supervision. **Frederic Khiami**: Project administration; formal analysis. **Gregoire Rougereau**: Methodology; validation. **Yoann Bohu**: Data curation; formal analysis. **Nicolas Lefevre**: Writing—review and editing; supervision; validation. **Alexandre Hardy**: Conceptualisation; data curation; methodology; writing—review and editing.

## CONFLICT OF INTEREST STATEMENT

The authors declare no conflicts of interest.

## ETHICS STATEMENT

Referenced under the number: COS‐RGDS‐2025‐07‐005‐HARDY‐A; IRB number (IRB00010835). Informed consent was obtained from all the patients.

## Data Availability

The data that support the findings of this study are available in the supplementary material of this article.

## References

[1] Acquadro C , Conway K , Hareendran A , Aaronson N . Literature review of methods to translate health‐related quality of life questionnaires for use in multinational clinical trials. Value Health. 2008;11(3):509–521. 10.1111/j.1524-4733.2007.00292.x 18179659

[2] Ardern CL , Glasgow P , Schneiders A , Witvrouw E , Clarsen B , Cools A , et al. 2016 consensus statement on return to sport from the first world congress in sports physical therapy, Bern. Br J Sports Med. 2016;50(14):853–864. 10.1136/bjsports-2016-096278 27226389

[3] Beaton DE , Bombardier C , Guillemin F , Ferraz MB . Guidelines for the process of cross‐cultural adaptation of self‐report measures. Spine. 2000;25(24):3186–3191. 10.1097/00007632-200012150-00014 11124735

[4] Bent NP , Wright CC , Rushton AB , Batt ME . Selecting outcome measures in sports medicine: a guide for practitioners using the example of anterior cruciate ligament rehabilitation. Br J Sports Med. 2009;43(13):1006–1012. 10.1136/bjsm.2009.057356 19224908

[5] Borloz S , Crevoisier X , Deriaz O , Ballabeni P , Martin RL , Luthi F . Evidence for validity and reliability of a French version of the FAAM. BMC Musculoskeletal Disord. 2011;12:40. 10.1186/1471-2474-12-40 PMC3045395 21303520

[6] Carcia CR , Martin RL , Drouin JM . Validity of the foot and ankle ability measure in athletes with chronic ankle instability. J Athl Train. 2008;43(2):179–183. 10.4085/1062-6050-43.2.179 PMC2267323 18345343

[7] Delahunt E , Bleakley CM , Bossard DS , Caulfield BM , Docherty CL , Doherty C , et al. Clinical assessment of acute lateral ankle sprain injuries (ROAST): 2019 consensus statement and recommendations of the international ankle consortium. Br J Sports Med. 2018;52(20):1304–1310. 10.1136/bjsports-2017-098885 29886432

[8] Delgado DA , Lambert BS , Boutris N , McCulloch PC , Robbins AB , Moreno MR , et al. Validation of digital visual analog scale pain scoring with a traditional paper‐based visual analog scale in adults. JAAOS: Glob Res Rev. 2018;2(3):e088. 10.5435/JAAOSGlobal-D-17-00088 PMC6132313 30211382

[9] Fermanian J . Validation des échelles d’évaluation en médecine physique et de réadaptation: comment apprécier correctement leurs qualités psychométriques. Annales de Réadaptation et de Médecine Physique. 2005;48(6):281–287. 10.1016/j.annrmp.2005.04.004 15923054

[10] Geerinck A , Beaudart C , Salvan Q , Van Beveren J , D'Hooghe P , Bruyère O , et al. French translation and validation of the Cumberland ankle Instability tool, an instrument for measuring functional ankle instability. Foot Ankle Surg. 2020;26(4):391–397. 10.1016/j.fas.2019.05.002 31118138

[11] Gribble PA , Delahunt E , Bleakley C , Caulfield B , Docherty C , Fourchet F , et al. Selection criteria for patients with chronic ankle instability in controlled research: a position statement of the International Ankle Consortium. Br J Sports Med. 2014;48(13):1014–1018. 10.1136/bjsports-2013-093175 24255768

[12] Gribble PA , Delahunt E , Bleakley CM , Caulfield B , Docherty CL , Fong DT‐P , et al. Selection criteria for patients with chronic ankle instability in controlled research: a position statement of the International Ankle Consortium. J Athl Train. 2014;49(1):121–127. 10.4085/1062-6050-49.1.14 PMC3917288 24377963

[13] Hansen CF , Obionu KC , Comins JD , Krogsgaard MR . Patient reported outcome measures for ankle instability. An analysis of 17 existing questionnaires. Foot Ankle Surg. 2022;28(3):288–293. 10.1016/j.fas.2021.04.009 34001448

[14] Hardy A , Freiha K , Moussa MK , Valentin E , Rauline G , Alvino K , et al. Use of ankle‐GO to assess and predict return to sport after lateral ankle reconstruction for chronic ankle instability. Orthop J Sports Med. 2025;13. 10.1177/23259671251322903 PMC11930476 40124190

[15] Hoch JM , Hartzell J , Kosik KB , Cramer RJ , Gribble PA , Hoch MC . Continued validation and known groups validity of the Quick‐FAAM: inclusion of participants with chronic ankle instability and ankle sprain copers. Phys Ther Sport. 2020;43:84–88. 10.1016/j.ptsp.2020.02.012 32135450

[16] Hoch JM , Legner JL , Lorete C , Hoch MC . The validity of the quick‐FAAM in patients seeking treatment for an acute or subacute foot or ankle health condition. J Sport Rehabil. 2017;26(3):jsr.2016‐0089. 10.1123/jsr.2016-0089 27633016

[17] Hoch JM , Powden CJ , Hoch MC . Reliability, minimal detectable change, and responsiveness of the Quick‐FAAM. Phys Ther Sport. 2018;32:269–272. 10.1016/j.ptsp.2018.04.004 29804692

[18] Hoch MC , Hoch JM , Houston MN . Development of the Quick‐FAAM: a preliminary shortened version of the foot and ankle ability measure for chronic ankle instability. Int J Athl Ther Train. 2016;21(4):45–50.

[19] Hung M , Baumhauer JF , Latt DL , Saltzman CL , SooHoo NF , Hunt KJ . Validation of PROMIS® physical function computerized adaptive tests for orthopaedic foot and ankle outcome research. Clin Orthop Relat Res. 2013;471(11):3466–3474. 10.1007/s11999-013-3097-1 PMC3792246 23749433

[20] Hung M , Nickisch F , Beals TC , Greene T , Clegg DO , et al. New paradigm for patient‐reported outcomes assessment in foot ankle research: computerized adaptive testing. Foot Ankle Int. 2012;33(8):621–626. 10.3113/FAI.2012.0621 22995227

[21] Lopes R , Decante C , Geffroy L , Brulefert K , Noailles T . Arthroscopic anatomical reconstruction of the lateral ankle ligaments: a technical simplification. Orthop Traumatol Surg Res. 2016;102(8S):S317–S322. 10.1016/j.otsr.2016.09.003 27692587

[22] Lutz C , Baverel L , Colombet P , Cournapeau J , Dalmay F , Lefevre N , et al. Pain after out‐patient vs. in‐patient ACL reconstruction: French prospective study of 1076 patients. Orthop Traumatol Surg Res. 2016;102(8S):S265–S270. 10.1016/j.otsr.2016.08.009 27687061

[23] Martin RL , Hutt DM , Wukich DK . Validity of the foot and ankle ability measure (FAAM) in diabetes mellitus. Foot Ankle Int. 2009;30(4):297–302. 10.3113/FAI.2009.0297 19356352

[24] Martin RL , Irrgang JJ . A survey of self‐reported outcome instruments for the foot and ankle. J Orthop Sports Phys Ther. 2007;37(2):72–84. 10.2519/jospt.2007.2403 17366962

[25] Martin RL , Irrgang JJ , Burdett RG , Conti SF , Swearingen JMV . Evidence of validity for the foot and ankle ability measure (FAAM). Foot Ankle Int. 2005;26(11):968–983. 10.1177/107110070502601113 16309613

[26] Martin RL , Irrgang JJ , Burdett RG , Conti SF , Swearingen JMV . Evidence of validity for the foot and ankle ability measure (FAAM). Foot Ankle Int. 2005;26(11):968–983. 10.1177/107110070502601113 16309613

[27] Mazaheri M , Salavati M , Negahban H , Sohani SM , Taghizadeh F , Feizi A , et al. Reliability and validity of the Persian version of foot and ankle ability measure (FAAM) to measure functional limitations in patients with foot and ankle disorders. Osteoarthritis Cartilage. 2010;18(6):755–759. 10.1016/j.joca.2010.03.006 20338253

[28] Nauck T , Lohrer H . Translation, cross‐cultural adaption and validation of the German version of the Foot and ankle ability measure for patients with chronic ankle instability. Br J Sports Med. 2011;45(10):785–790. 10.1136/bjsm.2009.067637 19955163

[29] Pioger C , Guillo S , Bouché P‐A , Sigonney F , Elkaïm M , Bauer T , et al. The ALR‐RSI score is a valid and reproducible scale to assess psychological readiness before returning to sport after modified Broström‐Gould procedure. Knee Surg Sports Traumatol Arthrosc. 2022;30(7):2470–2475. 10.1007/s00167-022-06895-7 PMC9206630 35079843

[30] Revelle W . psych: procedures for psychological, psychometric, and personality research. CRAN Contrib. Packag; 2007.

[31] Robin X , Turck N , Hainard A , Tiberti N , Lisacek F , Sanchez J‐C , et al. pROC: an open‐source package for R and S+ to analyze and compare ROC curves. BMC Bioinform. 2011;12:77. 10.1186/1471-2105-12-77 PMC3068975 21414208

[32] Roos EM , Brandsson S , Karlsson J . Validation of the foot and ankle outcome score for ankle ligament reconstruction. Foot Ankle Int. 2001;22(10):788–794. 10.1177/107110070102201004 11642530

[33] Ryu C . dlookr: tools for data diagnosis, exploration, transformation. CRAN Contrib. Packag; 2018.

[34] Sigonney F , Lopes R , Bouché P‐A , Kierszbaum E , Moslemi A , Anract P , et al. The ankle ligament reconstruction‐return to sport after injury (ALR‐RSI) is a valid and reproducible scale to quantify psychological readiness before returning to sport after ankle ligament reconstruction. Knee Surg Sports Traumatol Arthrosc. 2020;28(12):4003–4010. 10.1007/s00167-020-06020-6 PMC7669765 32356045

[35] Youden WJ . Index for rating diagnostic tests. Cancer. 1950;3(1):32–35. 10.1002/1097-0142(1950)3:1<32::aid-cncr2820030106>3.0.co;2-3 15405679

